# The Association of Soft Drink Consumption and the 24-Hour Movement Guidelines with Suicidality among Adolescents of the United States

**DOI:** 10.3390/nu14091870

**Published:** 2022-04-29

**Authors:** Bao-Peng Liu, Cun-Xian Jia, Shi-Xue Li

**Affiliations:** 1 Department of Epidemiology, School of Public Health, Cheeloo College of Medicine, Shandong University, Jinan 250012, China; liubaopeng@sdu.edu.cn; 2 Center for Suicide Prevention Research, Shandong University, Jinan 250012, China; 3 Centre for Health Management and Policy Research, School of Public Health, Cheeloo College of Medicine, Shandong University, Jinan 250012, China

**Keywords:** 24 h movement guidelines, soft drink, suicidality, adolescent

## Abstract

Background: Evidence is lacking for the association of the behaviors of the 24 h movement guidelines including sleep duration, physical activity, screen time, and soft drink consumption with suicidality among adolescents. Methods: Data were extracted from a national representative sample of Youth Risk Behavior Surveys (YRBS) in the United States from 2011 to 2019. Binary logistic regression models with complex sampling designs were used to explore the association of the recommendations of the 24 h movement guidelines and soft drink consumption with suicidality. Results: The total prevalence of suicidal ideation, suicide plan, suicide attempt, and suicide attempt with medical treatment was higher among adolescents who did not meet all the recommendations in the 24 h movement guidelines and had a higher level of soft drink consumption. Totally, not meeting all the recommendations of the 24 h movement guidelines was significantly associated with an increased risk of suicidal ideation (OR: 1.69, 95% CI: 1.30–2.19) and suicide plan (OR: 1.76, 95% CI: 1.34–2.33) compared with adolescents who meet all the recommendations. Soft drink consumption of ≥3 times/day was associated with an increased risk of suicidality including suicidal ideation, suicide plan, suicide attempt, and suicide attempt with medical treatment, regardless of sex. Soft drink consumption of ≥3 times/day was significantly associated with an increased risk of suicide attempt and suicide attempt with medical treatment, regardless of whether the recommendations of physical activity, screen time, and sleep duration were met. Conclusion: Age-appropriate sleep duration, no more than 2 h of screen time per day, at least 1 h of physical activity per day as contained in the 24 h movement guidelines and less than one soft drink consumption per day are good targets to prevent involvement in suicidality. More actions for intervening in the movement and dietary behaviors among adolescents are needed to maintain physical and mental health.

## 1. Introduction 

Suicide among adolescents brings a great burden of diseases worldwide and psychological pressure to the family [1,2]. Previous reports had identified the imperative of suicide, which is the fourth leading cause of death among 15–29 years old worldwide and the second leading cause of death among 10–34 years old in the United States (U.S.) [1,3,4]. Plenty of studies have materially identified recognized risk factors of suicide among adolescents such as depression [5], acute stressful events, chronic adversity in early life, familial, and genetic factors, and so on [2,5,6]. However, a perspective on lifestyle including dietary behaviors and physical activity should also be paid enough attention in adolescents, which are the important factors for physical and mental health [7,8,9,10].

In 2018, the World Health Organization (WHO) released some initiatives and guidelines about physical activity and set a goal to reduce physical inactivity by 15% by 2030 [11,12]. The guideline from the WHO recommends children and adolescents should do at least an average of 60 min per day of moderate to vigorous intensity, mostly aerobic, physical activity, and limit the amount of time spent being sedentary, particularly the amount of recreational screen time but without a precise threshold [12,13,14]. The Canadian 24 h movement guidelines for children and adolescents, which were released in 2016, are an integrative goal of physical activity, screen time, and sleep duration [15]. The detailed content of the 24 h movement guidelines was that an accumulation of at least 60 min per day of moderate to vigorous physical activity, uninterrupted 9–11 h of sleep per night for those aged 5–13 years and 8–10 h per night for those aged 14–17 years, and no more than 2 h per day of recreational screen time [15]. These guidelines adhere to the criteria of the WHO, specifically the threshold of sedentary behaviors, and add the recommendations of sleep duration, which agrees with the recommendations from the National Sleep Foundation [16] in children and adolescents. The integrative index could better reflect the effect of movement among children and adolescents compared with a single indicator and give us more evidence for protecting children and adolescents from adverse outcomes.

The prevalence of meeting all the recommendations of the 24 h movement guidelines reported in the previous studies is between 1.0% and 9.4% among children and adolescents [9,17,18,19,20,21,22]. What is more, the males and younger adolescents were reported to have a higher prevalence of meeting all the recommendations contained in the guidelines according to previous studies [9,18,20,23]. Previous studies have reported that meeting all the recommendations of the 24 h movement guidelines are associated with obesity or being overweight [20], global cognition [22], and mental health such as internalizing and externalizing behaviors [10], impulsivity [21], psychological distress [24], depressive symptoms [25,26], and anxiety [25]. Moreover, only one study, performed by Sampasa-Kanyinga et al. using the data from the Ontario Student Drug Use and Health Survey [9], reported the association of the 24 h movement guidelines and suicidality including suicide ideation, and suicide attempt by sex and age among children and adolescents. However, this study did not report the overall association of meeting the recommendations of the 24 h movement guidelines with suicidal ideation and suicide attempt, and lack of the association of the recommendations of the 24 h movement guidelines and suicide plan or suicide attempt with medical treatment, which are an important index of suicidality.

Soft drinks, especially the consumption of sweetened beverages, were found to be highly correlated to loneliness [27], sedentary behaviors [28], physical status such as unhealthy weight status [29,30] and early menarche [31], aggressive behaviors [32,33,34] and mental health [35,36,37,38,39], which are reported to be associated with suicidality among adolescents. Although previous studies also reported that soft drinks and sweetened drinks are directly associated with an increased risk of suicidality [32,40,41,42], most of the recent studies are from non-US and low- and middle-income countries [40,41,42]. Moreover, Solnick et al. used the national data of the Youth Risk Behavior Survey (YRBS) of the U.S. in 2009 to explore the associations among soft drinks, aggression, and suicidality [32]. However, this study did not examine the dose–response association and the evidence for the association in the recent 10 years is limited to the U.S.

More attention should be paid to the interactive association of movement and dietary behaviors with suicidality and mental health. A previous study used the data of YRBS in 2019 to explore the association of sleep duration, screen time, physical activity, and dietary behaviors (not including soft drinks) with suicidality [43]. Another study also using the data of YRBS in 2019 and latent class analysis tried to build a new variable of lifestyle including all the variables of the 24 h movement guidelines and dietary behaviors and explore their association with suicidality [44]. However, there are no studies to explore the association between soft drinks and suicidality by different recommendations of the 24 h movement guidelines, namely sleep duration, screen time, physical activity, and integrative index. In addition, the interactive association of not meeting the recommendations of the 24 h movement guidelines and more consumption of soft drinks with suicidality is also rarely reported.

This study used the data from YRBS of the U.S. from 2011 to 2019 and aimed to (1) document the weighted prevalence of suicidality including suicidal ideation, suicide plan, suicide attempt, and suicide attempt with medical treatment in total and by the level of soft drink consumption or different recommendations of the 24 h movement guidelines; (2) document the prevalence of meeting all, two, or one of the recommendations of the 24 h movement guidelines; (3) explore the weighted association of the 24 h movement guidelines and soft drink consumption with suicidality; (4) report the association of soft drink consumption and suicidality by different recommendations of the 24 h movement guidelines among adolescents of the U.S.

## 2. Methods

### 2.1. Design and Participants

The Youth Risk Behavior Surveillance System (YRBSS), developed in 1990 by the Centers for Disease Control and Prevention (CDC) in the U.S., aimed to monitor health-risk behaviors during childhood and early adolescence. YRBS, which was conducted every two years with different participants, was a national school-based survey of representative samples of 9th through to 12th-grade students. Employing a three-stage cluster sample design, YRBS included public and private schools in the 50 states and the District of Columbia. The first-stage sampling frame, namely, primary sampling units (PSUs), consisted of large-sized counties or groups of smaller, adjacent counties. In the second stage of sampling, selected schools from PSUs and one or two entire classes in each chosen school and in each of the grades 9–12 were randomly selected in the final stage of sampling. A weight based on sex, race/ethnicity, and school grade is applied to each record to adjust for student nonresponse and oversampling of Black and Hispanic students. The protocol of national YRBS was approved by the institutional review board of CDC and is publicly available. A self-administered computer-scannable questionnaire with anonymity was used with the voluntary procedure and parental permission. YRBS was a repeated cross-sectional database and reflected the status of high school in the U.S. More details about YRBS can be seen at the website [45] and previously published studies about YRBS [46,47]. In consideration of data integrity (the data on physical activity began in 2011), this study included the data of five recent 10-year surveys (2011, 2013, 2015, 2017, and 2019). The sample size of the five surveys was 15,425, 13,583, 15,624, 14,765, and 13,677, respectively, and a total of 73,074 adolescents were examined eventually in this study. 

### 2.2. Independent Variables

#### 2.2.1. Soft Drink Consumption

Soft drink consumption was measured by the question: *During the past 7 days, how many times did you drink a can, bottle, or glass of soda or pop, such as Coke, Pepsi, or Sprite? (do not count diet soda or diet pop)?* Response options included not drinking soda or pop during the past 7 days, drinking 1 to 3 times during the past 7 days, 4 to 6 times during the past 7 days, 1 time per day, 2 times per day, 3 times per day, 4 or more times per day. These were categorized into none, <1 time per day, 1–2 times per day, and 3 times or above per day in this study.

#### 2.2.2. The Recommendations of the 24 h Movement Guidelines

The recommendations of the 24 h movement guidelines included physical activity, screen time, and sleep duration. Physical activity was measured by the question: *During the past 7 days, how many days were you physically active for a total of at least 60 min per day? (Add up all the time you spent in any kind of physical activity that increased your heart rate and made you breathe hard some of the time).* Responses were dichotomized into 7 days (every day) and lower than 7 days. Screen time was extracted from two questions: *On an average school day how many hours do you (1) watch TV and (2) play video or computer games or use a computer for something that is not schoolwork?* After summing the time of the two questions, responses were dichotomized into above 2 h and 2 h or below. Sleep duration was measured by the question: *On the average school night, how many hours of sleep do you get?* Responses were dichotomized into adherence to the recommendations and not according to the guidelines (9–11 h per night for 11–13-year-olds; 8–10 h per night for 14–17-year-olds, or 7–9 h per night for those ≥18 years of age) [16,48].

Eventually, meeting the recommendations of the 24 h movement guidelines was assessed by two new variables: (1) meeting all the three criteria or not, and (2) meeting all the three criteria, meeting physical activity and screen time, meeting physical activity and sleep duration, meeting screen time and sleep duration, meeting physical activity only, meeting screen time only, meeting sleep duration only, and meeting none of the three criteria. The first variable was used to assess the prevalence of meeting all the recommendations of the 24 h movement guidelines and the association with suicidality. The second variable was used to check the distribution of meeting all and part recommendations of the 24 h movement guidelines.

### 2.3. Dependent Variables

Suicidality, namely, suicidal ideation, suicide plan, suicide attempt, and suicide attempt with medical treatment were the dependent variables in this study. Suicidal ideation was measured by the question: *During the past 12 months, did you ever seriously consider attempting suicide?* A suicide plan was measured by the question: *During the past 12 months, did you ever make a plan about how you would attempt suicide?* Responses for suicidal ideation and suicide plan were dichotomized into yes and no. Suicide attempt was measured by the question: *During the past 12 months, how many times did you actually attempt suicide?* Responses were dichotomized into none and 1 time or above. Suicide attempt with medical treatment was measured by the question: *If you attempted suicide during the past 12 months, did any attempt result in an injury, poisoning, or overdose that had to be treated by a doctor or nurse?* Responses were dichotomized into yes and no.

### 2.4. Covariates

#### 2.4.1. Demographic Factors

The demographic factors in this study included age, sex, race, and year of the survey. Age was categorized into 14 years old or below, 15 years old, 16 years old, 17 years old, and 18 years old or above. Race was ascertained with two questions. The first question was “Are you Hispanic or Latino?” and the second question was “What is your race?”. If the adolescents responded “yes” to the first question, they were identified as “Hispanic/Latino”. Otherwise, the second question would be asked with the response options of “White”, “Black or African American” and “others” (American Indian or Alaska Native, Asian, Native Hawaiian, or Other Pacific Islander). The year of the survey was used as a multinominal variable in this study.

#### 2.4.2. Weight Status

Age- and sex-specific Body Mass Index (BMI) was used to determine normal or underweight, overweight or obese in this study. The participants were considered overweight when the BMI percentile was at or above the 85th percentile and obese when the BMI percentile was at or above the 95th percentile for BMI by age and sex. The program and technical documentation for calculating and discriminating weight status could be seen on the website [49] and a previous study [50].

#### 2.4.3. Dietary Behaviors

Dietary behaviors in this study included vegetable, fruit, milk, and breakfast consumption. The responses of vegetables and fruit were dichotomized into one or more times per day and less than one time per day. The responses to milk consumption were dichotomized into one or more glasses per day and less than one glass per day. Breakfast consumption was categorized into daily and not daily. The question’s wording and detailed responses can be seen in Table S1.

#### 2.4.4. Depressive Symptoms 

Depressive symptoms were measured by the question: *During the past 12 months, did you ever feel so sad or hopeless almost every day for two weeks or more in a row that you stopped doing some usual activities?* The responses to this question were yes or no. This question is valid for depressive symptoms according to a previous study [44].

More details of the questions and responses associated with covariates, independent variables, and dependent variables can be seen in Table S1.

### 2.5. Statistical Analysis

The software of R version 4.1.0 was used to perform all the analyses in this study. A series of analyses related to complex sampling design was used to get valid point estimates and corresponding confidence intervals. The weighted prevalence of suicidality in total or by the recommendations of the 24 h movement guidelines and soft drink consumption was reported in this study. Pearson Chi-squared statistics with the second-order correction of the Rao–Scott Chi-square test [51] were used to explore the differences in the weighted prevalence of suicidality by the recommendations of the 24 h movement guidelines and soft drink consumption. The *p*-values for the differences were computed with a Satterthwaite approximation to the distribution and with denominator degrees of freedom as recommended by Thomas and Rao [52]. The confidence intervals of weighted prevalence were estimated by the methods proposed by Korn and Graubard [53]. Venn diagrams, which could display weight percentage clearly, were used to show the distributions of meeting the recommendations of the 24 h movement guidelines. Binary logistic regression models with a complex sampling design were used to show the association of meeting all the recommendations of the 24 h movement guidelines and soft drink consumption with suicidality, including suicidal ideation, suicide plan, suicide attempt, and suicide attempt with medical treatment after adjusting age, sex, race, survey year, weight status, depressive symptoms, and dietary behaviors including milk, fruit, vegetable, and breakfast consumption. Simultaneously, the association between soft drink consumption and suicidality by different recommendations of the 24 h movement guidelines was explored in this study.

Sensitivity analysis of missing data by multiple imputations by chained equations (MICE) was used to explore the stability of the associations among soft drink consumption, 24 h movement guidelines, and suicidality [46,54]. Sensitivity analysis of the association among the 24 h movement guidelines, soft drink consumption, and suicidality by omitting weight status and depressive symptoms was also performed in consideration of its confounding effect on the association. In addition, E-values were utilized to assess the sensitivity of potential unmeasured confounding results [55]. E-values for each exposure were calculated using an online calculator (website: www.evalue-calculator.com, accessed on 6 April 2022) with reporting the estimates and limits of corresponding 95% CI [56].

## 3. Results

### 3.1. Characteristics of Included Participants

Among 73,074 included participants, 87.9% of high-school students were 15-years-old or above. The ratio of boy/girl was 0.99:1 (36,108/36,497, others are missing). The proportions of White, Black/African American, Hispanic/Latino were 43.0%, 16.8%, and 27.1%. A total of 14.7% and 13.2% of the participants were overweight and obese. More details on the distribution of age, sex, race, and weight status, and unweighted proportions of dietary behaviors (soft drink, vegetable, fruit, milk, and breakfast consumption), the recommendations of the 24 h movement guidelines, depressive symptoms, and suicidality could be seen in Table S2.

### 3.2. The Weighted Prevalence of Suicidality by the Recommendations of the 24 h Movement Guidelines and Levels of Soft Drink Consumption

As shown in Table 1, the total prevalence of suicidal ideation, suicide plan, suicide attempt and suicide attempt with medical treatment was 17.3% (16.8–17.8%), 14.0% (13.5–14.5%), 8.1% (7.7–8.5%), and 2.6% (2.4–2.8%), respectively. The prevalence of suicidality in the group of meeting all the recommendations of the 24 h movement guidelines is significantly lower than in those not meeting all the recommendations. A lower prevalence of suicidality could also be seen in other recommendations, namely appropriate sleep duration, screen time ≤2 h/day, or physical activity ≥1 h/day.

The prevalence of suicidal ideation, suicide plan, suicide attempt, and suicide attempt with medical treatment associated with soft drink consumption of ≥3 time/day was 24.5% (22.9–26.2%), 20.9% (19.2–22.7%), 16.0% (14.5–17.5%) and 6.4% (5.3–7.5%). There was a significant difference in the prevalence of suicidal ideation, suicide plan, suicide attempt, and suicide attempt with medical treatment across different levels of soft drink consumption (all *p* < 0.001). As the frequency of soft drink consumption increased, the prevalence of suicidal ideation, suicide plan, suicide attempt, and suicide attempt with medical treatment increased.

### 3.3. The Weighted Prevalence of Meeting the Recommendations of the 24 h Movement Guidelines

**Figure 1:**
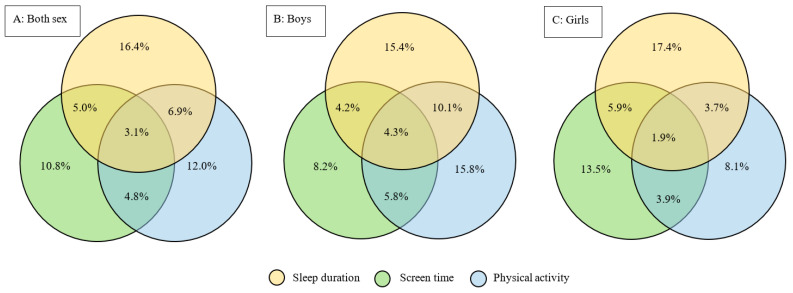
Venn diagrams showing the weighted prevalence of meeting all and part recommendations of the 24 h movement guidelines in total and by sex among the adolescents of the U.S.

The prevalence of meeting the relative recommendations of the 24 h movement guidelines can be seen in Figure 1. The prevalence of meeting all the recommendations contained in the guidelines was 3.1% in total, 4.3% for boys, and 1.9% for girls. Venn diagrams, shown in Figure 1, also gave us some findings on meeting two recommendations of the 24 h movement guidelines. The prevalence of only meeting the recommendations of sleep duration and screen time was 5.0%, 4.2%, and 5.9% in total, for boys, and for girls, respectively. The prevalence of only meeting the recommendations of sleep duration and physical activity was 6.9%, 10.1%, and 3.7% in total, for boys, and for girls, respectively. The prevalence of only meeting the recommendations of physical activity and screen time was 4.8%, 5.8%, and 3.9% in total, for boys, and for girls, respectively.

### 3.4. The Association of the 24 h Movement Guidelines and Soft Drink Consumption with Suicidality

Totally, not meeting all the recommendations of the 24 h movement guidelines was significantly associated with an increased risk of suicidal ideation (OR: 1.69, 95% CI: 1.30–2.19), and suicide plan (OR: 1.76, 95% CI: 1.34–2.32), compared with adolescents who meet all the recommendations. However, the association between meeting all the recommendations of the 24 h movement guidelines and suicide attempt (OR: 1.12, 95% CI: 0.74–1.68), and suicide attempt with medical treatment (OR: 1.04, 95% CI: 0.49–2.23) was not statistically significant. In the group of boys, similar results compared with the total estimates were found to be with a higher risk of suicide ideation (OR: 2.18, 95% CI: 1.51–3.13) and suicide plan (OR: 2.28, 95% CI: 1.56–3.34) associated with not meeting all the recommendations of the 24 h movement guidelines. The association of meeting all the recommendations of the 24 h movement guidelines with suicide attempt and suicide attempt with medical treatment was also not statistically significant among the boys. Additionally, the association between meeting all the recommendations and suicidality was not found to be statistically significant among the girls.

Soft drink consumption of 1–2 times/day was only found to be associated with an increased risk of suicidal ideation (OR: 1.15, 95% CI: 1.02–1.30) and suicide attempt (OR: 1.21, 95% CI: 1.04–1.41) in total, and suicide attempt (OR: 1.32, 95% CI: 1.09–1.59) and suicide attempt with medical treatment among the girls (OR: 1.47, 95% CI: 1.05–2.05).

Soft drink consumption of ≥3 times/day was associated with an increased risk of suicidality including suicidal ideation, suicide plan, suicide attempt, and suicide attempt with medical treatment whether in overall estimates or subgroup analysis by sex. Moreover, there was a linear dose–response relationship for soft drink consumption associated with an increased risk of suicidality among adolescents regardless of sex. More details can be seen in Table 2.

### 3.5. Subgroup Analyses of the Association between Soft Drink Consumption and Suicidality by Different Recommendations of the 24 h Movement Guidelines

**Figure 2:**
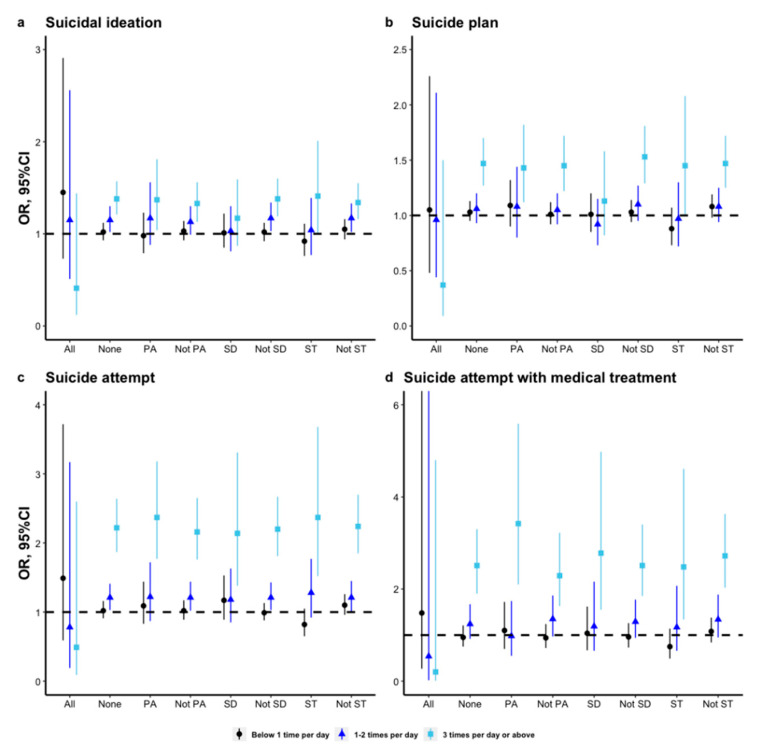
The association between soft drink consumption and suicidality by different recommendations of the 24 h movement guidelines among adolescents in the U.S. (OR: odds ratios, CI: confidence interval; All: meeting all the recommendations of the 24 h movement guidelines; None: meeting none of the recommendations of the 24 h movement guidelines; PA: meeting the recommendations of physical activity; Not PA: not meeting the recommendations of physical activity; SD: meeting the recommendations of sleep duration; Not PA: not meeting the recommendations of sleep duration; ST: meeting the recommendations of screen time; Not PA: not meeting the recommendations of screen time. The estimates of meeting all the recommended behaviors and not were adjusted for age, sex, race, survey year, weight status, depressive symptoms, and dietary behaviors including milk, fruit, vegetable, and breakfast consumption. The estimates of PA and not PA were adjusted for age, sex, race, survey year, weight status, depressive symptoms, and dietary behaviors including milk, fruit, vegetable, breakfast consumption, sleep duration, and screen time. The estimates of SD and not SD were adjusted for age, sex, race, survey year, weight status, depressive symptoms, and dietary behaviors including milk, fruit, vegetable, breakfast consumption, sleep duration, and physical activity. The estimates of ST and not ST were adjusted for age, sex, race, survey year, weight status, depressive symptoms, and dietary behaviors including milk, fruit, vegetable, breakfast consumption, sleep duration, and physical activity.).

Several findings emerged in the subgroup analyses. Firstly, the association between soft drink consumption and suicidality, regardless of suicidal ideation, suicide plan, suicide attempt, and suicide attempt with medical treatment was statistically significant among adolescents who were not meeting all the recommendations of the 24 h movement guidelines. The association was not found in adolescents who were meeting all the recommendations. Secondly, there was a significant interaction of not meeting the recommendation of screen time and soft drink consumption of <1 time/day on suicide plan and suicide attempt (Table S3). Thirdly, soft drink consumption of ≥3 times/day was significantly associated with an increased risk of suicide attempt and suicide attempt with medical treatment regardless of whether the adolescent was meeting the recommendations of physical activity, screen time, and sleep duration. Fourthly, the association between soft drink consumption and suicidal ideation and suicide plan was not statistically significant among adolescents who were not meeting all the recommendations of sleep duration, regardless of the frequency. More details of subgroup analyses can be seen in Figure 2.

### 3.6. Sensitivity Analysis

Multiple imputations by chained equations (MICE) were performed to explore the effect of missing data on the association among the recommendations of the 24 h movement guidelines, soft drink consumption, and suicidality. The estimates associated with the risks were slightly changed and revealed that the estimates were stable.

In addition, a sensitivity analysis (Table S4) of the association among the 24 h movement guidelines, soft drink consumption, and suicidality by omitting weight status and depressive symptoms was also performed. Although the effects were enhanced and lower levels of soft drink consumption were statistically associated with increased risk of suicidality, the association of the 24 h movement guidelines and soft drink consumption with suicidality was similar to previous estimates.

The E-values (Table S5) were relatively large, particularly for the association with three times per day or above of soft drink consumption. Our findings show that any unobserved confounder could be adequate to fully explain away these effect estimates and to move the CIs to null, while a weak confounder could not do so.

## 4. Discussions

### 4.1. Recommendations of the 24 h Movement Guidelines and Suicidality

To our knowledge, this is the first study to report the prevalence of meeting the recommendations of the 24 h movement guidelines taking advantage of the integrated index of physical activity, screen time, and age-appropriate sleep duration in the study of YRBS. Although Zhu et al. [20], using data from the 2016–2017 National Survey of Children’s Health (NSCH) of the U.S., reported a higher prevalence (9.4%) of meeting all the recommendations of the 24 h movement guidelines, this study reported a comparable prevalence (3.1%, 95% CI: 2.8–3.4%) with previous studies [9,17,18,19,21,22]. Similar to most previous studies [9,18,20,23], the boys had a higher prevalence of meeting all the recommendations contained in the guidelines in this study. Despite all this, children and adolescents worldwide were reported to have a low prevalence of meeting all the recommendations of the 24 h movement guidelines.

Previous studies usually explored the association between one variable in an adolescent’s lifestyle such as sedentary behaviors [57,58], screen time [59], sleep duration [60], physical activity [61], and suicidality. This study used the integrated index, namely the 24 h movement guidelines, which could reflect the movement of adolescents effectively to explore the association with suicidality. The findings in this study were consistent with a previous study from Canada [9], reporting that not meeting all the recommendations of the 24 h movement guidelines could significantly increase the risk of suicidal ideation and suicide attempt only among the boys. Moreover, our study added some evidence that the associations for suicide plan and suicide attempt with medical treatment and the total estimates for the associations among adolescents.

This study reported that there was no statistically significant association between meeting the 24 h movement guidelines and suicidality, regardless of suicidal ideation, suicide plan, suicide attempt, and suicide attempt with medical treatment among the girls, which was somewhat consistent with a previous study [9]. Several mechanisms may explain the differences by sex. Firstly, the girls have a lower prevalence of alcohol use [62], which might mediate the associations between meeting movement guidelines and suicidality [63]. Furthermore, the girls have a lower prevalence of suicide attempt [6] and a lower prevalence of meeting the recommendations of the 24 h movement guidelines [9,18,20,23], which might not have enough statistical power to detect the associations.

### 4.2. Soft Drink Consumption and Suicidality

The proportion of consuming no soft drink being 23.5% in this study is similar to previous studies [34,40,41]. Although different cut-offs for the levels of soft drink consumption were used in previous studies, only a higher frequency of soft drink consumption, namely above one time per day was associated with increased risk of suicidality including suicidal ideation, suicide plan, and suicide attempt in the previous studies [32,40,41,42]. This study also added some new evidence for the association between high levels of soft drink consumption and suicide attempt with medical treatment. Moreover, this study also had similar conclusions that the association of soft drink consumption with suicide attempt would be not changed in the subgroup analysis of sex with a previous study [42]. What is more, a significant association, regardless of sex, was also found in other behaviors of suicidality including suicidal ideation, suicide plan, and suicide attempt with medical treatment.

Although a previous study using the data of YRBS in 2009 reported soft drink consumption daily or above was associated with suicidality [32], this study added to the evidence of the recent 10 years for the linear dose–response relationship among adolescents in the U.S. The estimated risk in this study was fully adjusted by the dietary behaviors and depressive symptoms, which were not performed in previous studies. The mechanism from soft drink consumption to suicidality could be explained by mental problems. Many previous studies have examined that soft drink consumption was associated with depressive symptoms [35,36,37,38,39], which is highly related to suicidality among adolescents. Moreover, a high-sugar diet in adolescents was highly related to neuroinflammation, depressive-like behavior [64], stress-driven, emotional and addictive behaviors [65], which might be related to suicidality. In addition, previous studies reported that soft drink consumption was related to obesity or being overweight [29,30], which might cause inflammation among depressed patients [66]. This path could also be a reason for suicidality among adolescents in consideration of the positive effect of being overweight, inflammation, and depression on suicidality [67,68]. A high-sugar diet could increase anxiety-like and depressive-like behavior [69,70], decrease cognitive performance [71], and chronic psychological stress, development of metabolic syndrome (MetS), and behavioral impairment [72] among mice. This evidence from animals could provide some mediating paths from a high-sugar diet to suicidality.

### 4.3. Interactive Association of the 24 h Movement Guidelines and Soft Drink Consumption with Suicidality

Previous studies also tried to explore the combined association of lifestyle including dietary behaviors and movement behaviors with suicidality by the methods of exploring the individual association for included variables or using latent class analysis [39,40], limited studies focus on the interactive association with suicidality. It is worth noting that a significant association between any level of soft drink consumption and suicidality was not found in the group that met all the recommendations of the 24 h movement guidelines in this study. It might be explained that the negative effect of soft drinks could be decreased when adolescents have good habits of movement. In other groups including those not meeting all the recommendations and meeting the recommendations of sleep duration, screen time, and physical activity contained in the 24 h movement guidelines, the higher level of soft drink consumption, namely above two times per day, was significantly associated with suicidality. It is worth noting the important role of controlling the consumption of soft drinks and keeping suitable movement among adolescents.

### 4.4. Strengths and Limitations

This study had some strengths. Firstly, this study used national school-based data from representative samples of 9th through to 12th-grade students to emphasize a linear dose–response relationship between soft drink consumption and suicidality, namely suicidal ideation, suicide plan, suicide attempt, and suicide attempt with medical treatment. In addition, this is the first study using YRBS to report the recommendations of the 24 h movement guidelines and explore their association with suicidality. Furthermore, this is also the first study to explore the association of soft drink consumption and suicidality with different recommendations of the 24 h movement guidelines.

Some limitations are worth mentioning in this study. Firstly, a causal relationship is not able to be confirmed given the cross-sectional design. More cohort studies are needed to explore the relationship in the future. Secondly, all the questions were self-reported, and recall bias and information bias were unavoidable. Movement variables such as sleep duration, physical activity, and screen time were not measured by wearable devices, which might lead to information bias. Thirdly, soft drinks in this study did not include energy drinks, which were not collected in YRBS, which might bring some effect on the association. Fourthly, owing to the design of YRBS focusing on schools, findings are not suitable for extending to the entire population of adolescents. Lastly, some important socioeconomic factors such as family income, occupation of parents, and dietary habits of parents were not included in this database, which could be associated with confounding factors. Although the database included some factors such as alcohol and cigarette use, it was not able to be included as the covariates owing to the limited sample size in the subgroup of girls or meeting all the recommendations of the 24 h movement guidelines.

## 5. Conclusions

The present study supported the evidence that not meeting the recommendations of the 24 h movement guidelines and a high level of soft drink consumption could increase the risk of suicidality. It is implicated that the association of soft drink consumption with suicidality was not statistically significant when adolescents meet all the recommendations of the 24 h movement guidelines. These findings emphasize the importance of age-appropriate sleep duration, limited screen time (≤2 h/day), and appropriate physical activity (≥1 h/day) contained in the 24 h movement guidelines, and less consumption of soft drinks for preventing suicidality among adolescents. Relevant departments, schools, and families should formulate corresponding measures to ensure these beneficial behaviors and prevent at-risk adolescents from adverse behaviors.

## Figures and Tables

**Table 1:** The weighted prevalence of meeting the recommendations of the 24 h movement guidelines and suicidality by levels of soft drink consumption among adolescents of the U.S.

Variables	Suicidality, % (95% CI)			
	Suicidal Ideation	Suicide Plan	Suicide Attempt	Suicide Attempt with Medical Treatment
Total	17.3 (16.8–17.8)	14.0 (13.5–14.5)	8.1 (7.7–8.5)	2.6 (2.4–2.8)
The recommendations of the 24 h movement guidelines				
Appropriate sleep duration ^a^	11.4 (10.8–12.0)	9.5 (9.0–10.1)	5.2 (4.7–5.7)	1.5 (1.3–1.8)
Inappropriate sleep duration ^a^	20.2 (19.6–20.8)	16.3 (15.6–16.9)	9.2 (8.7–9.7)	2.9 (2.6–3.2)
*p* for difference	<0.001	<0.001	<0.001	<0.001
Screen time ≤2 h/day	14.7 (13.8–15.6)	11.9 (11.0–12.8)	7.1 (6.3–7.9)	2.4 (2.0–2.8)
Screen time >2 h/day	18.1 (17.5–18.6)	14.7 (14.2–15.3)	8.3 (7.9–8.6)	2.5 (2.3–2.7)
*p* for difference	<0.001	<0.001	0.005	0.496
Physical activity ≥1 h/day	13.0 (12.3–13.8)	10.9 (10.2–11.6)	6.3 (5.8–6.8)	2.1 (1.8–2.3)
Physical activity <1 h/day	18.8 (18.2–19.4)	15.2 (14.6–15.8)	8.7 (8.2–9.2)	2.7 (2.4–3.0)
*p* for difference	<0.001	<0.001	<0.001	<0.001
Meeting all the recommendations	6.8 (5.4–8.2)	4.7 (3.6–5.9)	3.5 (2.3–4.7)	1.2 (0.3–2.0)
Not meeting all the recommendations	17.8 (17.2–18.3)	14.4 (13.9–15.0)	8.0 (7.6–8.5)	2.5 (2.3–2.7)
*p* for difference	<0.001	<0.001	<0.001	0.036
Soft drink consumption				
None	15.3 (14.5–16.2)	12.5 (11.7–13.3)	6.6 (6.0–7.2)	2.1 (1.7–2.4)
<1 time/day	16.8 (16.1–17.4)	13.5 (12.9–14.2)	7.3 (6.8–7.7)	2.1 (1.8–2.3)
1–2 times/day	18.5 (17.3–19.7)	14.4 (13.3–15.5)	8.5 (7.7–9.4)	2.8 (2.3–3.3)
≥3 time/day	24.5 (22.9–26.2)	20.9 (19.2–22.7)	16.0 (14.5–17.5)	6.4 (5.3–7.5)
*p* for difference	<0.001	<0.001	<0.001	<0.001

^a^ Appropriate sleep duration means 9–11 h/day for adolescents aged 11–13, 8–10 h/day for adolescents aged 14–17, and 7–9 h/day for adolescents aged above 18 years. CI: confidence interval.

**Table 2:** The association of the 24 h movement guidelines and soft drink consumption with suicidality among adolescents of the U.S.

Variables	Suicidal Ideation, OR (95% CI) ^a^	Suicide Plan, OR (95% CI) ^a^	Suicide Attempt, OR (95% CI) ^a^	Suicide attempt with medical treatment, OR (95% CI) ^a^
Total				
24 h movement guidelines				
Meeting all the recommendations	Reference	Reference	Reference	Reference
Not meeting all the recommendations	1.69 (1.30–2.19) ***	1.76 (1.34–2.32) ***	1.12 (0.74–1.68)	1.04 (0.49–2.23)
Soft drink consumption				
None	Reference	Reference	Reference	Reference
<1 time/day	1.03 (0.94–1.12)	1.03 (0.95–1.13)	1.03 (0.92–1.16)	0.96 (0.77–1.21)
1–2 times/day	1.15 (1.02–1.30) *	1.06 (0.93–1.20)	1.21 (1.04–1.41) *	1.25 (0.93–1.67)
≥3 times/day	1.37 (1.20–1.55) ***	1.45 (1.26–1.68) ***	2.20 (1.86–2.61) ***	2.49 (1.90–3.27) ***
*p* for trend	<0.001	<0.001	<0.001	<0.001
Boy				
24 h movement guidelines				
Meeting all the recommendations	Reference	Reference	Reference	Reference
Not meeting all the recommendations	2.18 (1.51–3.13) ***	2.28 (1.56–3.34) ***	1.68 (0.87–3.23)	0.80 (0.27–2.37)
Soft drink consumption				
None	Reference	Reference	Reference	Reference
<1 time/day	0.97 (0.83–1.13)	1.01 (0.87–1.18)	0.79 (0.61–1.02)	0.58 (0.38–0.89) *
1–2 times/day	1.11 (0.91–1.36)	1.01 (0.83–1.24)	0.99 (0.75–1.32)	0.87 (0.53–1.43)
≥3 times/day	1.40 (1.15–1.71) ***	1.52 (1.22–1.90) ***	2.09 (1.59–2.76) ***	2.58 (1.60–4.16) ***
*p* for trend	<0.001	0.001	<0.001	<0.001
Girl				
24 h movement guidelines				
Meeting all the recommendations	Reference	Reference	Reference	Reference
Not meeting all the recommendations	1.30 (0.89–1.90)	1.36 (0.90–2.07)	0.78 (0.44–1.35)	1.53 (0.71–3.30)
Soft drink consumption				
None	Reference	Reference	Reference	Reference
<1 time/day	1.04 (0.94–1.17)	1.03 (0.93–1.15)	1.15 (0.99–1.33)	1.17 (0.92–1.49)
1–2 times/day	1.16 (0.99–1.36)	1.09 (0.91–1.29)	1.32 (1.09–1.59) **	1.47 (1.05–2.05) *
≥3 times/day	1.29 (1.08–1.55) **	1.36 (1.13–1.63) **	2.13 (1.73–2.62) ***	2.19 (1.64–2.94) ***
*p* for trend	0.003	0.004	<0.001	<0.001

^a^ All the estimates in these tables were adjusted for age, sex, race, survey year, weight status, depressive symptoms, and dietary behaviors including milk, fruit, vegetable, and breakfast consumption. Sex was not adjusted in the stratified models. OR: odds ratio, CI: confidence interval, *** *p* < 0.001, ** *p* < 0.01, * *p* < 0.05.

## Data Availability

The data can be downloaded from https://www.cdc.gov/healthyyouth/data/yrbs/data.htm/.

## Supplementary Materials

The following supporting information can be downloaded at: https://www.mdpi.com/article/10.3390/nu14091870/s1, Table S1: Question-wording and details for included variables; Table S2: Characteristics of included variables among youth risk behavior surveys by survey year (2011–2019); Table S3: Interactive association of soft drink consumption and the recommendations of the 24 h movement guidelines with suicidality; Table S4: Sensitivity analysis of the association among 24-h movement guidelines, soft drink consumption and suicidality by omitting weight status and depressive symptoms; Table S5: E-value analysis for the association among soft drink consumption, 24-h movement guideline, and suicidality.
