# Supplementary material for: The Association of Soft Drink Consumption and the 24-Hour Movement Guidelines with Suicidality among Adolescents of the United States
Source: Nutrients. 2022 Apr 29;14(9):1870. doi: 10.3390/nu14091870 (PMC9100874; doi:10.3390/nu14091870)
Supplement: Supplementary file 1 [file nutrients-14-01870-s001.zip › nutrients-1625825-supplementary.pdf]

Table S1: Question wording and details for included variables.

Variables	Question	Response options	Coding for analysis
**Physical activity**	*During the past 7 days, on how many days were you physically active for a total of at least 60 minutes per day? (Add up all the time you spent in any kind of physical activity that increased your heart rate and made you breathe hard some of the time)*	0 days, 1 day, 2 days, 3 days, 4 days, 5 days, 6 days, 7 days	Responses were dichotomized into 7-days and <7-days
**Screen time**	*On an average school day how many hours do you . . . (1) watch TV? and (2) play video or computer games or use a computer for something that is not schoolwork?*	No, less than 1 hour per day, 1 hour per day, 2 hours per day, 3 hours per day, 4 hours per day, 5 or more hours per day	After summing the time of the two questions, responses were dichotomized into >2-hours and 2-hours or below
**Sleep duration**	*On the average school night, how many hours of sleep do you get?*	4 or less hours, 5 hours, 6 hours, 7 hours, 8 hours, 9 hours, 10 or more hours	Responses were dichotomized into adherence to the recommendations and not according to the guidelines (9–11 h per night for 11–13-year-old; 8–10 h per night for 14–17-year-old, or 7–9 h per night for those ≥18 years of age)
**Breakfast consumption**	*During the past 7 days, on how many days did you eat breakfast?*	0 days, 1 day, 2 days, 3 days, 4 days, 5 days, 6 days, 7 days	Responses were categorized into not daily, and daily

**Vegetable consumption**	*During the past 7 days, how many times did you eat (1) green salad? (2) potatoes (Do not count French fries, fried potatoes, or potato chips? (3) carrots? (4) other vegetables? (Do not count green salad, potatoes, or carrots.)*	I did not eat… during the past 7 days, 1 to 3 times during the past 7 days, 4 to 6 times during the past 7 days, 1 time per day, 2 times per day, 3 times per day, 4 or more times per day	After summing the frequency of the four questions, responses were dichotomized into one or more times per day and less than one time per day
**Fruit consumption**	*During the past 7 days, how many times did you (1) drink 100% fruit juices such as orange juice, apple juice, or grape juice? (Do not count punch, Kool-Aid, sports drinks, or other fruit-flavored drinks.) (2) eat fruit? (Do not count fruit juice)*	I did not eat/drink… during the past 7 days, 1 to 3 times during the past 7 days, 4 to 6 times during the past 7 days, 1 time per day, 2 times per day, 3 times per day, 4 or more times per day	After summing the frequency of the two questions, responses were dichotomized into one or more times per day and less than one time per day
**Milk consumption**	*During the past 7 days, how many glasses of milk did you drink? (Count the milk you drank in a glass or cup, from a carton, or with cereal. Count the half pint of milk served at school as equal to one glass)*	I did not drink milk during the past 7 days, 1 to 3 glasses during the past 7 days, 4 to 6 glasses during the past 7 days, 1 glass per day, 2 glasses per day, 3 glasses per day, 4 or more glasses per day	Responses were dichotomized into one or more glasses per day and less than one glass per day
**Soft drink consumption**	*During the past 7 days, how many times did you drink a can, bottle, or glass of soda or pop, such as Coke, Pepsi, or Sprite? (Do not count diet soda or diet pop.)*	I did not drink soda or pop during the past 7 days, 1 to 3 times during the past 7 days, 4 to 6 times during the past 7 days, 1 time per day, 2 times per day, 3 times per day, 4 or more times per day	Responses were categorized into none, <1 time per day, 1-2 times per day, and 3 times or above per day.

**Depressive symptoms**	*During the past 12 months, did you ever feel so sad or hopeless almost every day for two weeks or more in a row that you stopped doing some usual activities?*	Yes or no	Responses were dichotomized into yes and no.
**Suicidal ideation**	*During the past 12 months, did you ever seriously consider attempting suicide?*	Yes or no	Responses were dichotomized into yes and no.
**Suicide plan**	*During the past 12 months, did you ever make a plan about how you would attempt suicide?*	Yes or no	Responses were dichotomized into yes and no.
**Suicide attempt**	*During the past 12 months, how many times did you actually attempt suicide?*	0 times, 1 time, 2 or 3 times, 4 or 5 times, 6 or more times	Responses were dichotomized into 0 and 1+times.
**Suicide attempt with medical treatment**	*If you attempted suicide during the past 12 months, did any attempt result in an injury, poisoning, or overdose that had to be treated by a doctor or nurse?*	Yes or no	Responses were dichotomized into yes and no.

Table S2: Characteristics of included variables among youth risk behavior surveys by survey year (2011–2019).

Variables	Total, n (%) [b]	Survey year, n (%)				
		2011	2013	2015	2017	2019
**Total number**	73,074 (100)	15,425 (21.1)	13,583 (18.6)	15,624 (21.4)	14,765 (20.2)	13,677 (18.7)
**Demographic factors**						
**Age (years old)**						
≤14	8574 (11.7)	1629 (10.6)	1412 (10.4)	1744 (11.2)	2003 (13.6)	1786 (13.1)
15	17,444 (23.9)	3470 (22.5)	3098 (22.8)	3817 (24.4)	3586 (24.3)	3473 (25.4)
16	18,613 (25.5)	4061 (26.3)	3203 (23.6)	4033 (25.8)	3688 (25.0)	3628 (26.5)
17	17,940 (24.6)	3921 (25.4)	3473 (25.6)	3833 (24.5)	3611 (24.5)	3102 (22.7)
≥18	10,145 (13.9)	2282 (14.8)	2320 (17.1)	2131 (13.6)	1796 (12.2)	1616 (11.8)
**Sex**						
Boy	36,108 (49.4)	7656 (49.6)	6950 (51.2)	7749 (49.6)	7112 (48.2)	6641 (48.6)
Girl	36,497 (49.9)	7708 (50.0)	6621 (48.7)	7757 (49.6)	7526 (51.0)	6885 (50.3)
**Race**						
White	31,398 (43.0)	6171 (40.0)	5449 (40.1)	6849 (43.8)	6261 (42.4)	6668 (48.8)
Black or African American	12,263 (16.8)	2767 (17.9)	2993 (22.0)	1667 (10.7)	2796 (18.9)	2040 (14.9)
Hispanic/Latino	19,828 (27.1)	4627 (30.0)	3395 (25.0)	5121 (32.8)	3647 (24.7)	3038 (22.2)
All other races	7819 (10.7)	1545 (10.0)	1428 (10.5)	1629 (10.4)	1724 (11.7)	1493 (10.9)
**Weight status**						
Normal or underweight	46,083 (63.1)	10,069 (65.3)	8729 (64.3)	9822 (62.9)	9051 (61.3)	8412 (61.5)
Overweight	10,769 (14.7)	2258 (14.6)	2064 (15.2)	2365 (15.1)	2149 (14.6)	1933 (14.1)
Obesity	9657 (13.2)	1958 (12.7)	1787 (13.2)	2171 (13.9)	1946 (13.2)	1795 (13.1)
**Dietary behaviors**						
**Breakfast consumption**						
Daily	21,883 (29.9)	4387 (28.4)	4875 (35.9)	4800 (30.7)	4135 (28.0)	3686 (27.0)
Not daily	40,822 (55.9)	7628 (49.5)	8448 (62.2)	9056 (58.0)	7783 (52.7)	7907 (57.8)
**Vegetable consumption**						
One or more times per day	40,625 (55.6)	8579 (55.6)	8036 (59.2)	9043 (57.9)	8026 (54.4)	6941 (50.7)
Less than one time per day	27,812 (38.1)	5862 (38.0)	5191 (38.2)	6136 (39.3)	5807 (39.3)	4816 (35.2)
**Fruit consumption**						
One or more times per day	42,797 (58.6)	9229 (59.8)	8453 (62.2)	9493 (60.8)	8455 (57.3)	7167 (52.4)
Less than one time per day	27,353 (37.4)	5425 (35.2)	4869 (35.8)	5808 (37.2)	5889 (39.9)	5362 (39.2)

Variables	Total, n (%) [b]	Survey year, n (%)				
		2011	2013	2015	2017	2019
**Milk consumption**						
One or more glasses per day	21,353 (29.2)	5161 (33.5)	5081 (37.4)	4860 (31.1)	3599 (24.4)	2652 (19.4)
Less than one glass per day	38,829 (53.1)	7177 (46.5)	8199 (60.4)	8355 (53.5)	8261 (55.9)	6837 (50.0)
**Soft drink consumption**						
None	17,136 (23.5)	2875 (18.6)	2961 (21.8)	3734 (23.9)	3978 (26.2)	3588 (26.2)
<1 time/day	36,101 (49.4)	7429 (48.2)	6773 (49.9)	8145 (52.1)	7676 (52.0)	6078 (44.4)
1–2 times/day	93,12 (12.8)	2377 (15.4)	1994 (14.7)	2147 (13.7)	1653 (11.2)	1141 (8.3)
≥3 times/day	6070 (8.3)	1623 (10.5)	1596 (11.7)	1266 (8.1)	997 (6.8)	588 (4.3)
**Recommendations of 24 h movement guideline**						
**Sleep duration [a]**						
Meeting recommendations	19,712 (27.0)	4251 (27.6)	4277 (31.5)	4574 (29.3)	3311 (22.4)	3299 (24.1)
Not meeting recommendations	43,952 (60.1)	7892 (51.2)	7987 (58.8)	9897 (63.3)	8433 (57.1)	9743 (71.2)
**Screen time**						
≤2 h/day	16,203 (22.2)	3372 (21.9)	2582 (19.0)	3536 (22.6)	3517 (23.8)	3196 (23.4)
>2 h/day	53,491 (73.2)	11,577 (75.1)	10,626 (78.2)	11,515 (73.7)	10,264 (69.5)	9509 (69.5)
**Physical activity**						
≥1 h/day	17,969 (24.6)	4058 (26.3)	3622 (26.7)	3893 (24.9)	3442 (23.3)	2954 (24.6)
<1 h/day	53,092 (72.7)	10,990 (71.2)	9688 (71.3)	11,352 (72.7)	10,796 (73.1)	10,266 (75.1)
**Depressive symptoms or suicidality**						
**Depressive symptoms**						
Yes	22,969 (31.4)	4537 (29.4)	4086 (30.1)	4789 (30.7)	4631 (31.4)	4926 (36.0)
no	49,198 (67.3)	10,732 (69.6)	9409 (69.3)	10,666 (68.3)	9896 (67.0)	8495 (62.1)
**Suicidal ideation**						
Yes	12,695 (17.4)	2424 (15.7)	2259 (16.6)	2808 (18.0)	2571 (17.4)	2633 (19.3)
no	59,513 (81.4)	12,869 (83.4)	11,232 (82.7)	12,626 (80.8)	11,982 (81.2)	10,804 (79.0)
**Suicide plan**						
Yes	10,401 (14.2)	2015 (13.1)	1874 (13.8)	2331 (14.9)	2030 (13.7)	2151 (15.7)
no	61,466 (84.1)	13,263 (86.0)	11,611 (85.5)	12,810 (82.0)	12,511 (84.7)	11,271 (82.4)
**Suicide attempt**						
Yes	5301 (7.3)	1179 (7.6)	1015 (7.5)	1203 (7.7)	837 (5.7)	1067 (7.8)
no	53,968 (73.9)	12,335 (80.0)	10,967 (80.7)	11,364 (72.7)	9849 (66.7)	9453 (69.1)

Variables	Total, n (%) [b]	Survey year, n (%)				
		2011	2013	2015	2017	2019
**Suicide attempt with medical treatment**						
Yes	1582 (2.2)	348 (2.3)	324 (2.4)	399 (2.6)	286 (1.9)	225 (1.6)
no	54,827 (75.0)	12,611 (81.8)	11,426 (84.1)	11,932 (76.4)	10,334 (70.0)	8524 (62.3)

a: The 24 h movement guidelines for adolescents of sleep duration is 9–11 hour/night for 11–13 years, 8–10 hour/night for 14–17 years, and 7–9 hour/night for 18 years or above years.

b: Missing values were existed in the reported variables.

Table S3: Interactive association of soft drink consumption and the recommendations of 24 h movement guidelines with suicidality.

**Interaction**	**Odds Ratio, 95% CI [a]**			
	**Suicidal ideation**	**Suicide plan**	**Suicide attempt**	**Suicide attempt with medical treatment**
**All the recommendations of 24 h movement guidelines**				
Not meeting * soft drink consumption (<1 time/day)	0.76 (0.42–1.38)	0.98 (0.50–1.92)	0.63 (0.22–1.78)	0.33 (0.06–1.73)
Not meeting * soft drink consumption (1–2 time/day)	0.98 (0.48–2.02)	1.03 (0.51–2.08)	1.07 (0.32–3.62)	0.83 (0.11–6.47)
Not meeting * soft drink consumption (≥3 times/day)	2.48 (0.81–7.58)	2.81 (0.87–9.07)	2.96 (0.65–13.37)	4.08 (0.42–39.63)
**Physical activity**				
Not meeting * soft drink consumption (<1 time/day)	1.05 (0.83–1.34)	0.96 (0.78–1.18)	0.95 (0.69–1.31)	0.91 (0.52–1.56)
Not meeting * soft drink consumption (1–2 time/day)	0.97 (0.71–1.33)	0.98 (0.73–1.31)	1.01 (0.68–1.49)	1.44 (0.77–2.67)
Not meeting * soft drink consumption (≥3 times/day)	0.97 (0.70–1.34)	1.05 (0.80–1.36)	0.93 (0.65–1.33)	0.72 (0.39–1.31)
**Sleep duration**				
Not meeting * soft drink consumption (<1 time/day)	0.97 (0.81–1.17)	1.00 (0.82–1.22)	0.84 (0.63–1.13)	0.90 (0.54–1.52)
Not meeting * soft drink consumption (1–2 time/day)	1.13 (0.90–1.43)	1.18 (0.91–1.52)	1.05 (0.74–1.49)	1.15 (0.62–2.13)
Not meeting * soft drink consumption (≥3 times/day)	1.14 (0.84–1.57)	1.34 (0.93–1.92)	1.05 (0.65–1.69)	0.98 (0.52–1.85)
**Screen time**				
Not meeting * soft drink consumption (<1 time/day)	1.14 (0.92–1.40)	1.24 (1.01–1.51)	1.32 (1.00–1.73)	1.41 (0.91–2.19)
Not meeting * soft drink consumption (1–2 time/day)	1.09 (0.81–1.47)	1.11 (0.81–1.52)	0.95 (0.64–1.39)	1.09 (0.57–2.08)
Not meeting * soft drink consumption (≥3 times/day)	0.90 (0.63–1.30)	0.98 (0.68–1.42)	0.93 (0.58–1.51)	1.03 (0.51–2.08)

a: The estimates of interactive item related to meeting all the recommendations were adjusted for age, gender, race, survey year, weight status, and dietary behaviors including milk, fruit, vegetable, breakfast consumption and depressive symptoms. The estimates of interactive item related to PA were adjusted for age, gender, race, survey year, weight status, and dietary behaviors including milk, fruit, vegetable, depressive symptoms, breakfast consumption, sleep duration and screen time. The estimates of interactive item related to SD were adjusted for age, gender, race, survey year, weight status, and dietary behaviors including milk, fruit, vegetable, breakfast consumption, depressive symptoms, sleep duration and physical activity. The estimates of interactive item related to ST were adjusted for age, gender, race,

survey year, weight status, and dietary behaviors including milk, fruit, vegetable, breakfast consumption, depressive symptoms, sleep duration and physical activity. OR, odds ratio, CI: confidence interval.

Table S4. Sensitivity analysis of the association among 24 h movement guidelines, soft drink consumption and suicidality by omitting weight status and depressive symptoms.

**Variables**	**Suicidal ideation, OR (95%CI)** [a]	**Suicide plan, OR (95%CI)** [a]	**Suicide attempt, OR (95%CI)** [a]	**Suicide attempt with medical treatment, OR (95%CI)** [a]
**24 h movement guidelines**				
Meeting all the recommendations	Reference	Reference	Reference	Reference
Not meeting all the recommendations	2.14 (1.69–2.70) ***	2.45 (1.90–3.16) ***	1.58 (1.08–2.30) *	1.34 (0.64–2.82)
**Soft drink consumption**				
None	Reference	Reference	Reference	Reference
<1 time/day	1.13 (1.04–1.22) **	1.12 (1.04–1.21) **	1.14 (1.03–1.26) *	1.07 (0.86–1.32)
1–2 times/day	1.33 (1.18–1.50) ***	1.24 (1.10–1.41) ***	1.43 (1.24–1.65) ***	1.49 (1.12–1.97) **
≥3 times/day	2.01 (1.78–2.28) ***	2.06 (1.79–2.37) ***	3.21 (2.75–3.75) ***	3.88 (2.99–5.04) ***
P for trend	<0.001	<0.001	<0.001	<0.001

a: All the estimates in this tables were adjusted for age, sex, race, survey year, weight status, depressive symptoms, and dietary behaviors including milk, fruit, vegetable, and breakfast consumption. OR: odds ratio, CI: confidence interval, ***:$P < 0.001$, **: $P < 0.01$, *: $P < 0.05$.

Table S5. E-value analysis for the association among soft drink consumption, 24 h movement guideline, and suicidality

Variables	Suicidal ideation, E-value (confidence limit) [a]	Suicide plan, E-value (confidence limit) [a]	Suicide attempt, E-value (confidence limit) [a]	Suicide attempt with medical treatment, E-value (confidence limit) [a]
**24 h movement guidelines**				
Meeting all the recommendations	Reference	Reference	Reference	Reference
Not meeting all the recommendations	1.92 (1.54)	2.92 (2.01)	1.49 (1.00)	1.24 (1.00)
**Soft drink consumption**				
None	Reference	Reference	Reference	Reference
<1 time/day	1.14 (1.00)	1.21 (1.00)	1.21 (1.00)	1.25 (1.00)
1–2 times/day	1.35 (1.00)	1.31 (1.00)	1.71 (1.24)	1.81 (1.00)
≥3 times/day	1.62 (1.42)	2.26 (1.83)	3.82 (3.12)	4.42 (3.21)

a: All the estimates in this tables were adjusted for age, sex, race, survey year, weight status, depressive symptoms, and dietary behaviors including milk, fruit, vegetable, and breakfast consumption.
